# Seroprevalence of IgA and IgM antibodies to *Bordetella pertussis* in healthy Japanese donors: Assessment for the serological diagnosis of pertussis

**DOI:** 10.1371/journal.pone.0219255

**Published:** 2019-07-01

**Authors:** Rei Fumimoto, Nao Otsuka, Hajime Kamiya, Tomimasa Sunagawa, Keiko Tanaka-Taya, Kazunari Kamachi, Keigo Shibayama

**Affiliations:** 1 Department of Bacteriology II, National Institute of Infectious Diseases, Musashimurayama, Tokyo, Japan; 2 Department of Pediatrics, St Marianna University School of Medicine, Kawasaki, Japan; 3 Infectious Disease Surveillance Center, National Institute of Infectious Diseases, Tokyo, Japan; Universidad Nacional de la Plata, ARGENTINA

## Abstract

Pertussis is a human respiratory infection caused by the gram-negative bacterium, *Bordetella pertussis*. To evaluate the pertussis burden and vaccine efficacy, diagnosis and epidemiological surveillance should be based on accurate and valid diagnostic methods. Recently, the serodiagnostic tests Novagnost Bordetella pertussis IgA and IgM were approved in Japan for pertussis diagnostics. Although the anti-pertussis toxin (PT) IgG assay has been used for pertussis diagnosis worldwide, little is known about the anti-*B*. *pertussis* IgA and IgM assays. In this study, serum samples from 460 healthy donors were examined to determine the seroprevalence of anti-*B*. *pertussis* IgA and IgM in a Japanese population, and its correlation with donor age. Our data demonstrated that anti-*B*. *pertussis* IgA and IgM are positively and negatively correlated with age (r = 0.27, r = -0.37; P < 0.001, respectively). Age-specific analysis revealed high titers of anti-*B*. *pertussis* IgA in adults (46–50 years), while anti-*B*. *pertussis* IgM titers were high in schoolchildren (6–10, 11–15 years). When applying the arbitrary cut-off values for these ages, 17.6% and 39.5% of healthy donors were interpreted as pertussis-positive or indeterminate with anti-*B*. *pertussis* IgA (46–50 years) and IgM (11–15 years) titers, respectively. Overall, our findings indicated that the Novagnost Bordetella pertussis IgA and IgM testing could be greatly affected by subject age, limiting its value for pertussis diagnosis.

## Introduction

Pertussis, caused by the gram-negative bacterium *Bordetella pertussis*, is a highly contagious respiratory disease that is particularly severe in young infants. Although the primary strategy for preventing pertussis is vaccination, large outbreaks have recently occurred in developed countries with high vaccination rates, indicating that pertussis can affect people of any age, including vaccinated adolescents and adults [1–4]. To evaluate the pertussis burden and vaccine efficacy, diagnosis and epidemiological surveillance should be conducted based on accurate and valid diagnostic methods. Serology has been performed in many countries with different vaccination backgrounds using IgG-based enzyme-linked immunosorbent assays (ELISAs); however, the results varied between studies, making it challenging to comprehend and compare the actual pertussis burden [5]. International effort has been made to overcome the difficulties associated with pertussis serology based on the following recommendations: i) purified non-detoxified pertussis toxin (PT) should be used as antigen and ii) the results should be quantitatively expressed in international units (IU/ml) by using World Health Organization (WHO) international standards [6, 7]. However, the standardization of serological assays remains challenging, as numerous assays, which have mostly been employed for single-point evaluation in recent years, target other *B*. *pertussis* antigens and immunoglobulin classes [8–13].

In Japan, pertussis surveillance was conducted through the National Epidemiological Surveillance of Infectious Diseases (NESID) system at approximately 3000 pediatric sentinel clinics through 2017 [14]. However, such a pediatric sentinel surveillance does not fully reflect the trends in pertussis infection in the whole population; moreover, only clinically diagnosed cases have been reported. Therefore, to ensure reliable epidemiological data for pertussis in Japan, the ordinance for the Infectious Disease Control Law was extensively revised in January 2018 and pertussis was designated as a notifiable disease, requiring laboratory-confirmed diagnosis [15]. Because of the increased requirement for accurate diagnostics, the serodiagnostic tests Novagnost Bordetella pertussis IgA and IgM were recently approved for use as *in vitro* diagnostic assays by the Japanese regulatory agencies. Currently, 11.2% of pertussis cases are reported based on the results by these assays [16]. These serological testing kits are available for the detection of antibodies to *B*. *pertussis* in Japan. Although the anti-PT IgG assay has been used for pertussis diagnosis worldwide, little is known about the anti-*B*. *pertussis* IgA and IgM assays.

The aim of this study was to determine the seroprevalence of anti-*B*. *pertussis* IgA and IgM in a Japanese population using Novagnost Bordetella pertussis IgA and IgM kits for its diagnostic assessment. A total of 460 serum samples from healthy Japanese donors were examined for age-specific antibody distributions. The diagnostic interpretations based on the anti-*B*. *pertussis* IgA and IgM titers observed were evaluated with the current cut-off values, according to the age groups. The correlations of these antibody titers with those of anti-PT IgG and anti-filamentous haemagglutinin (FHA) IgG were also determined to understand the effects of age on the anti-*B*. *pertussis* IgA and IgM distributions.

## Materials and methods

### Serum samples

A total of 460 human serum samples were obtained from the National Serum Reference Bank of the National Institute of Infectious Diseases (Tokyo, Japan). The National Serum Reference Bank stores the serum remnants of the National Epidemiological Surveillance of Vaccine-Preventable Diseases. The population-based seroepidemiological profiles were regularly characterized by this project for selected vaccine-preventable infectious diseases in Japan [17]. Healthy blood donors were randomly selected among those from whom survey officers were able to obtain informed consent without biases in regard to age, sex, and geographic area in 2015–2016 (age range: 1–60 years; median age: 30.5 years). Personal or clinical information of the donors was not collected. The donors were recruited, for example, from local government officials and their families and from people attending routine health check-ups including those conducted among school children. All blood donors provided written informed consent. The protocol was reviewed and approved by the Human Ethics Committee of the National Institute of Infectious Diseases (Approval No. 846).

### ELISAs

Novagnost Bordetella pertussis IgA and Novagnost Bordetella pertussis IgM ELISA kits (Siemens Healthcare Diagnostics GmbH, Munich, Germany) were used to measure anti-*B*. *pertussis* IgA and IgM antibody titers. The ELISA plates were coated with purified PT and FHA mixed antigens for IgA antibody and inactivated *B*. *pertussis* whole cells for IgM antibody. However, the information about the content and purity of each antigen was not obtained from the vendor. ELISAs were performed manually according to the manufacturer’s instructions. Microtiter plates were read at a wavelength of 450 nm using a Multiskan FC Microplate Photometer (Thermo Scientific, Waltham, MA, USA) with a reference wavelength of 650 nm. The kits contained negative, cut-off, and positive sera, and serum IgA and IgM antibody titers were represented in Novagnost units (NTU) using the following formula: (mean absorbance value of sample × 10)/ (mean absorbance value of cut-off serum) = NTU. Anti-*B*. *pertussis* IgA and IgM titers were interpreted according to the cut-off values provided by the manufacturer as negative (<8.5 NTU), indeterminate (8.5–11.5 NTU) and positive (>11.5 NTU).

### Statistical analysis

Statistical analyses were conducted using GraphPad Prism version 7.0 (GraphPad, Inc., San Diego, CA, USA). Statistical comparisons of antibody titers among age groups were performed by the Kruskal-Wallis test followed by Dunn’s multiple comparisons test. A P value of <0.05 was considered to indicate a statistically significant difference.

## Results

### Age-specific prevalence of anti-*B*. *pertussis* IgA and IgM antibodies

**Fig 1:**
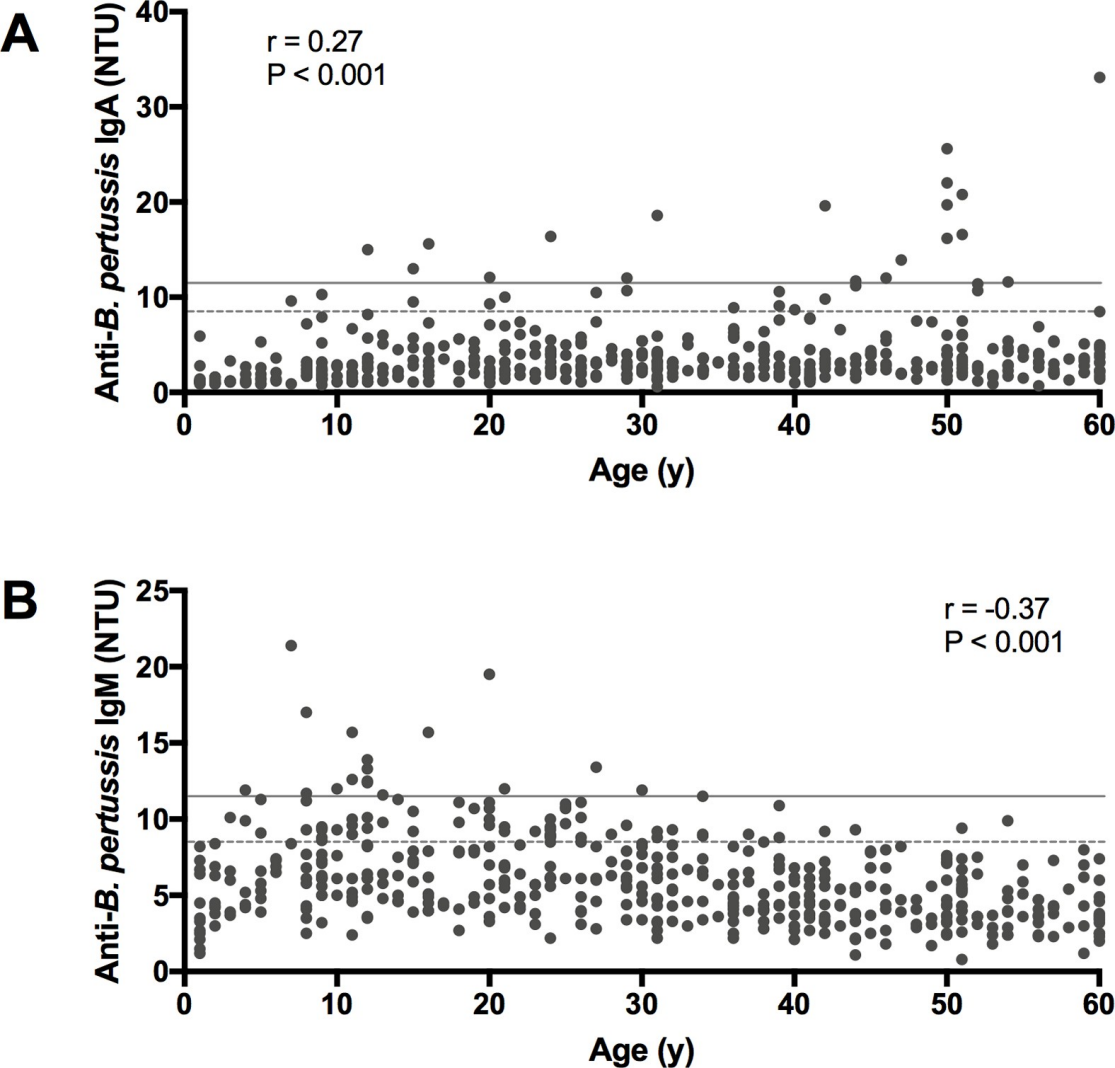
Seroprevalence of anti-*B*. *pertussis* IgA and IgM in healthy Japanese donors. (A) anti-*B*. *pertussis* IgA, (B) anti-*B*. *pertussis* IgM; Spearman correlation coefficients and P values are given in the panels. The arbitrary cut-off values for positive and indeterminate titer values are indicated by straight gray lines (11.5 NTU) and dashed lines (8.5 NTU), respectively.

The coefficient of variations (CVs) for the anti-*B*. *pertussis* IgA and IgM assays were calculated using the control sera; the ranges of within and between run-reproducibilities were 1.5–11.0% and 7.7–19.1%, respectively, which meet the conditions provided by the manufacturer’s instruction. Fig 1 shows the seroprevalence of anti-*B*. *pertussis* IgA and IgM in the Japanese healthy population. Anti-*B*. *pertussis* IgA titers ranged from 0.6 to 33.1 NTU and were weakly associated with age (r = 0.27, P < 0.001 in Fig 1A). Whereas anti-*B*. *pertussis* IgA titers showed a positive correlation with age, anti-*B*. *pertussis* IgM titers were negatively correlated with age in a narrower titer range (r = -0.37, P < 0.001; range 0.8–21.4 NTU in Fig 1B). To determine the age-specific distribution of antibodies, serum samples were divided into 12 age groups. Compared to the 1–5 years age group, all the other 11 age groups showed significantly higher mean titers of anti-*B*. *pertussis* IgA (Table 1). The highest mean titer of anti-*B*. *pertussis* IgA was observed in the 46–50 years age group (mean ± SD, 6.0 ± 6.3 in Table 1), and it was 3.8-fold higher than that in the lowest age group (1–5 years; mean ± SD, 1.6 ± 1.1). For anti-*B*. *pertussis* IgM, the 6–10, 11–15, and 21–25 years age groups showed significantly higher IgM mean titers than the reference age group of 1–5 years (Table 1). The lowest and highest mean titers of anti-*B*. *pertussis* IgM were observed in the 56–60 and 11–15 years age groups, respectively (mean ± SD, 4.1 ± 1.6 and 7.9 ± 3.2 in Table 1). The manufacturer recommends using both anti-*B*. *pertussis* IgA and IgM assays for the patients in the early phase of infection. However, no significant correlation was found between anti-*B*. *pertussis* IgA and IgM antibody titers among the 460 healthy donors (r = 0.07, P = 0.163).

**Table 1:** Seroprevalence of anti-*B*. *pertussis* IgA and IgM titers among different age groups.

Age group (years)	n	Anti-*B*. *pertussis* IgA titers		Anti-*B*. *pertussis* IgM titers	
		Mean ± SD	P value^a^	Mean ± SD	P value^a^
1–5	37	1.6 ± 1.1	-	5.5 ± 2.6	-
6–10	39	2.9 ± 2.2	0.007	7.7 ± 3.5	0.019
11–15	43	3.6 ± 3.0	<0.001	7.9 ± 3.2	0.006
16–20	34	4.1 ± 3.2	<0.001	7.3 ± 3.6	ns
21–25	39	4.0 ± 2.8	<0.001	7.2 ± 2.4	0.042
26–30	38	4.0 ±2.5	<0.001	6.7 ± 2.5	ns
31–35	35	3.4 ± 2.9	<0.001	5.9 ± 2.3	ns
36–40	42	3.8 ± 2.4	<0.001	5.2 ± 2.0	ns
41–45	43	4.0 ± 3.5	<0.001	4.8 ± 1.9	ns
46–50	34	6.0 ± 6.3	<0.001	4.5 ± 1.8	ns
51–55	39	4.7 ± 4.2	<0.001	4.7 ± 2.0	ns
56–60	37	4.1 ± 5.1	<0.001	4.1 ± 1.6	ns

NOTE. Antibody titers are represented in Novagnost units (NTU).

^a^ Dunn’s multiple comparison test; the 1–5 years age group was used as a control group for comparison. Significant at P value < 0.05, ns: not significant.

### Diagnostic results of Novagnost Bordetella pertussis IgA and IgM assays

Table 2 shows the diagnostic results of the Novagnost Bordetella pertussis IgA and IgM assays obtained for healthy donors using the arbitrary cut-off values recommended by the manufacture. Regarding the anti-*B*. *pertussis* IgA based diagnosis, the positivity rate and the proportion of positive and indeterminate titers among the 460 samples assessed were 4.1% and 7.6%, respectively. The age-specific proportion of positive and intermediate titers of anti-*B*. *pertussis* IgA peaked at 46–50 years age group with a value of 17.6%. When giving a diagnosis based on anti-*B*. *pertussis* IgM titers, the positivity rate was 3.7%, however the proportion of positive and indeterminate titers increased to 17.2% among 460 samples. Overall, the proportion of positive and indeterminate titers with anti-*B*. *pertussis* IgM assay were higher in the 6–25 years age group than that in other age groups. The 11–15 years age group showed a particularly high proportion (39.5%) of positive and indeterminate titers with anti-*B*. *pertussis* IgM assay.

**Table 2:** Diagnostic results based on the arbitrary cut-off values according to age group.

		Anti-*B*. *pertussis* IgA					Anti-*B*. *pertussis* IgM				
Age group (years)	n	Neg.	Ind.	Pos.	Pos.+Ind. (%)		Neg.	Ind.	Pos.	Pos.+Ind. (%)	
1–5	37	37	0	0	0	(0.0)	32	4	1	5	(13.5)
6–10	39	37	2	0	2	(5.1)	27	8	4	12	(30.8)
11–15	43	40	1	2	3	(7.0)	26	10	7	17	(39.5)
16–20	34	31	1	2	3	(8.8)	25	7	2	9	(26.5)
21–25	39	37	1	1	2	(5.1)	24	14	1	15	(38.5)
26–30	38	35	2	1	3	(7.9)	30	6	2	8	(21.1)
31–35	35	34	0	1	1	(2.9)	29	6	0	6	(17.1)
36–40	42	38	4	0	4	(9.5)	39	3	0	3	(7.1)
41–45	43	39	2	2	4	(9.3)	41	2	0	2	(4.7)
46–50	34	28	0	6	6	(17.6)	34	0	0	0	(0.0)
51–55	39	34	2	3	5	(12.8)	37	2	0	2	(5.1)
56–60	37	35	1	1	2	(5.4)	37	0	0	0	(0.0)
Total	460	425	16	19	35	(7.6)	381	62	17	79	(17.2)

NOTE: The antibody titers are represented in Novagnost unit (NTU) as negative (Neg., <8.5 NTU), indeterminate (Ind., 8.5–11.5 NTU), and positive (Pos., >11.5 NTU).

## Discussion

In the present study, anti-*B*. *pertussis* IgA and IgM antibody titers were measured in the 460 serum samples obtained from healthy Japanese donors. Anti-*B*. *pertussis* IgA antibody titers showed a positive correlation with age, while IgM antibody titers showed a negative correlation with age. Age-specific distribution analyses revealed that high mean titers were observed in adults (46–50 years) for anti-*B*. *pertussis* IgA, and in schoolchildren (6–10, 11–15 years) for anti-*B*. *pertussis* IgM. When applying the arbitrary cut-off values for these ages, 17.6% and 39.5% of healthy donors were interpreted as pertussis-positive or indeterminate with anti-*B*. *pertussis* IgA (46–50 years) and IgM (11–15 years) titers, respectively.

Previously, serological assays combining different antigens and immunoglobulins have been largely evaluated for their diagnostic performance, and anti-PT IgG assays calibrated to a reference standard were shown to be superior to IgA and IgM assays [12]. It was also reported that the mixed antigens such as PT plus FHA or whole cells drastically decreased the analytical parameters. In other studies, IgM-based assays showed poor diagnostic performance, particularly in those using whole-cell ELISA antigen, because of their low specificity [7, 18]. FHA is considered to have non-specific cross-reactivity with other *Bordetella* species or bacteria, e.g., non-encapsulated *H*. *influenzae*, *M*. *pneumoniae*, *C*. *pneumoniae*, which means that an increase in the anti-FHA antibody is not specific for *B*. *pertussis* infection [19–21]. In the present study, the coated ELISA antigens in Novagnost Bordetella pertussis IgA or IgM kits are a mixture of PT and FHA or *B*. *pertussis* whole cell lysate; therefore, being assumed to have the non-specific responses. The evaluation study of commercial ELISA kits in Germany showed that the Novagnost anti-*B*. *pertussis* IgA kit has a sensitivity and specificity of 72% and 75%, respectively [18]; thus, the anti-*B*. *pertussis* IgM kit was speculated to have a much lower diagnostic accuracy. In S1 Table, the correlation analyses showed that overall anti-*B*. *pertussis* IgA and IgM had only a negligible or weak correlation against anti-PT and FHA IgG (r = 0.05~0.41), while their correlations were slightly higher against anti-FHA IgG. This observation also suggests the existence of non-specific responses in both assays, especially the anti-*B*. *pertussis* IgM assay.

Although it remains unclear how the anti-*B*. *pertussis* IgM is elevated in schoolchildren, the high titers of anti-*B*. *pertussis* IgA in adults may partially be explained by asymptomatic *B*. *pertussis* infection. Our results showed that anti-*B*. *pertussis* IgA titers increased with age (Fig 1), and a significant correlation between anti-*B*. *pertussis* IgA and anti-PT IgG, a reference antibody for pertussis, was found in the 41–45 years age group (r = 0.61, P<0.001 in S2 Table). The datasets presented in S2 Table can also be found in our recent publication [22]. While high correlations should be found between anti-PT IgA and IgG in the serum of pertussis patients [23], the correlation degree was still moderate in this study, indicating the interference of non-specific responsivity caused by the FHA antigen. In previous studies as well, the positive correlations between age and the titers of anti-PT IgA were reported, and the cumulative effect of *B*. *pertussis* exposure was attributed to age [24–26]. The accumulated experiences of *B*. *pertussis* exposure are likely to be the cause of the increased anti-*B*. *pertussis* IgA levels in adults. *B*. *pertussis* natural infection induces both IgA and IgG development to *B*. *pertussis*, whereas vaccination only induces IgG to this bacterium [24, 27]. Therefore, high titers of anti-*B*. *pertussis* IgA accompanying anti-PT IgG in the 41–45 years age group may suggest a recent asymptomatic infection with *B*. *pertussis*. Moreover, our previous analyses on antibodies to PT also indicated the circulation of *B*. *pertussis* among healthy Japanese adults [28].

Of additional concerns for the application of Novagnost Bordetella pertussis IgA and IgM kits are the arbitrary unit (NTU) and cut-off values used for evaluating antibody titers. WHO has established the international standard pertussis antiserum (NIBSC code: 06/140) for data comparison among different serological assays [29, 30]. The 06/140 serum was assigned the international unit (IU) for IgG and IgA against PT, FHA, and pertactin (PRN). By using Novagnost Bordetella pertussis IgA and IgM kits, we confirmed that 335 IU/ml of anti-PT IgG of the 06/140 serum was corresponded to 18.4 NTU and 7.1 NTU of anti-*B*. *pertussis* IgA and IgM, respectively. However, the unit conversion from NTU to IU/ml is generally impossible because of the mixed ELISA antigens of the kits, which prevent data comparison with other validated assays. Furthermore, the current cut-off values need to be reviewed since our results showed the high proportion of pertussis positive or indeterminate titers in healthy donors with the anti-*B*. *pertussis* IgA and IgM. In general, the cut-off value would be raised to reduce the number of false-positives, but this can be achieved only when the antibody titers distribute uniformly across all ages in healthy individuals. It should be noted that the anti-PT IgG was significantly increased in the 1–2 years age group, indicating vaccine immunity, but a significant age-correlation was not found among healthy donors in 3–60 years (r = -0.001, P = 0.98 in S2 Table) [22]. A seroepidemiological survey in Japan currently uses a cut-off of ≥100 EU/ml anti-PT IgG for single-point diagnosis, and the positivity rate in this study was 1.8% in 3–60 years. Meanwhile, in the cases of anti-*B*. *pertussis* IgA and IgM, a higher cut-off will reduce the false-positives for some ages; it will, however, also lead to the excessively low sensitivity and high false-negative results for the other ages according to the sloping antibody titers observed in healthy individuals. Therefore, it seems difficult to establish the new cut-offs to enhance the diagnostic accuracy of these kits. One limitation of our study was that anti-*B*. *pertussis* IgA and IgM were only determined retrospectively in healthy donors. Improvement in the understanding of IgA and IgM responses in pertussis patients should contribute to the further evaluation of the anti-*B*. *pertussis* IgA and IgM assays.

In conclusion, our results demonstrated that anti-*B*. *pertussis* IgA and IgM titers in healthy Japanese donors are positively and negatively correlated with age, respectively. Age-specific analysis revealed high titers of anti-*B*. *pertussis* IgA were detected in adults (46–50 years), while anti-*B*. *pertussis* IgM titers were high mostly in schoolchildren (6–10, 11–15 years). The diagnostic results with the arbitrary cut-off values indicated that a large proportion of healthy individuals in these age groups was pertussis-positive or indeterminate with anti-*B*. *pertussis* IgA and IgM titers. Our findings indicate that the Novagnost Bordetella pertussis IgA and IgM testing can be greatly affected by subject age, which implies its limited value for pertussis diagnosis. To increase the accuracy of diagnosis and seroepidemiological assessment for *B*. *pertussis* infections, the credibility of the assays should be reconsidered.

## Supporting information

S1 Table Antibody titer-correlations in healthy Japanese donors according to age group. The anti-*B*. *pertussis* IgA and IgM titers of 460 healthy Japanese donors were evaluated the antibody correlations between anti-PT or FHA IgG according to age group. Correlations were determined with the nonparametric Spearman correlation test. Correlation coefficients were interpreted according to previously proposed stratifications: |r| < 0.1, negligible; 0.1 < |r| < 0.39, weak; 0.4 < |r| < 0.69, moderate; 0.7 < |r| < 0.89, strong; 0.9 < |r| < 1.0, very strong [31].
(DOCX)

S2 Table Seroprevalence of anti-PT IgG and anti-FHA IgG titers among different age groups. The anti-PT IgG and anti-FHA IgG titer of 460 healthy Japanese donors were measured in different age groups. The 1–2 years age group was used as a control group for antibody comparison. Titers of IgG to PT and FHA were measured using in-house ELISA with purified PT (Kaketsuken, Co. Ltd., Kumamoto, Japan) and FHA (Enzo Life Sciences, Farmingdale, NY, USA) as coated antigens. The IgG-based ELISAs were performed as previously described, except that serum samples were heated at 56°C for 30 min [28]. The IgG titers were converted from ELISA units (EU/ml) to international units (IU/ml) using the Pertussis Antiserum (human) 1^st^ IS-WHO International Standard 06/140 (NIBSC, UK). The datasets presented in S2 Table can also be found in our recent publication [22].
(DOCX)
