# Supplementary material for: Seroprevalence of IgA and IgM antibodies to Bordetella pertussis in healthy Japanese donors: Assessment for the serological diagnosis of pertussis
Source: PLoS One. 2019 Jul 1;14(7):e0219255. doi: 10.1371/journal.pone.0219255 (PMC6602288; doi:10.1371/journal.pone.0219255)
Supplement: S1 Table — The anti-B. pertussis IgA and IgM titers of 460 healthy Japanese donors were evaluated the antibody correlations between anti-PT or FHA IgG according to age group. Correlations were determined with the nonparametric Spearman correlation test. Correlation coefficients were interpreted according to previously proposed stratifications: |r| < 0.1, negligible; 0.1 < |r| < 0.39, weak; 0.4 < |r| < 0.69, moderate; 0.7 < |r| < 0.89, strong; 0.9 < |r| < 1.0, very strong [31]. (DOCX) [file pone.0219255.s001.docx]

**S1 Table**. **Antibody titer-correlations in healthy Japanese donors according to age group**

| Age group (years) | n |  | Anti-*B. pertussis* IgA | | | | |  | Anti-*B. pertussis* IgM | | | | |
| --- | --- | --- | --- | --- | --- | --- | --- | --- | --- | --- | --- | --- | --- |
|  |  |  | vs Anti-PT IgG | |  | vs Anti-FHA IgG | |  | vs Anti-PT IgG | |  | vs Anti-FHA IgG | |
|  |  |  | r | P value^a^ |  | r | P value^a^ |  | r | P value^a^ |  | r | P value^a^ |
| 1–5 | 37 |  | 0.04 | ns |  | 0.35 | 0.033 |  | -0.17 | ns |  | 0.08 | ns |
| 6–10 | 39 |  | 0.19 | ns |  | 0.56 | < 0.001 |  | 0.35 | 0.028 |  | 0.20 | ns |
| 11–15 | 43 |  | 0.48 | 0.001 |  | 0.50 | < 0.001 |  | 0.38 | 0.011 |  | 0.30 | 0.049 |
| 16–20 | 34 |  | 0.61 | < 0.001 |  | 0.66 | < 0.001 |  | 0.25 | ns |  | 0.52 | 0.002 |
| 21–25 | 39 |  | 0.34 | 0.034 |  | 0.29 | ns |  | -0.08 | ns |  | -0.07 | ns |
| 26–30 | 38 |  | 0.41 | 0.010 |  | 0.59 | < 0.001 |  | -0.09 | ns |  | 0.09 | ns |
| 31–35 | 35 |  | 0.18 | ns |  | 0.30 | ns |  | 0.20 | ns |  | 0.35 | 0.037 |
| 36–40 | 42 |  | 0.09 | ns |  | 0.42 | < 0.001 |  | -0.36 | 0.019 |  | -0.07 | ns |
| 41–45 | 43 |  | 0.61 | < 0.001 |  | 0.70 | < 0.001 |  | 0.15 | ns |  | 0.18 | ns |
| 46–50 | 34 |  | 0.47 | 0.005 |  | 0.68 | < 0.001 |  | -0.003 | ns |  | 0.07 | ns |
| 51–55 | 39 |  | 0.30 | ns |  | 0.49 | 0.002 |  | 0.20 | ns |  | 0.14 | ns |
| 56–60 | 37 |  | 0.17 | ns |  | 0.34 | 0.038 |  | 0.03 | ns |  | 0.31 | ns |
| Total | 460 |  | 0.26 | < 0.001 |  | 0.41 | < 0.001 |  | 0.05 | ns |  | 0.23 | <0.001 |

^a^ Significant at P value < 0.05; ns: not significant.
