# Supplementary material for: Seroprevalence of IgA and IgM antibodies to Bordetella pertussis in healthy Japanese donors: Assessment for the serological diagnosis of pertussis
Source: PLoS One. 2019 Jul 1;14(7):e0219255. doi: 10.1371/journal.pone.0219255 (PMC6602288; doi:10.1371/journal.pone.0219255)
Supplement: S2 Table — The anti-PT IgG and anti-FHA IgG titer of 460 healthy Japanese donors were measured in different age groups. The 1–2 years age group was used as a control group for antibody comparison. Titers of IgG to PT and FHA were measured using in-house ELISA with purified PT (Kaketsuken, Co. Ltd., Kumamoto, Japan) and FHA (Enzo Life Sciences, Farmingdale, NY, USA) as coated antigens. The IgG-based ELISAs were performed as previously described, except that serum samples were heated at 56°C for 30 min [28]. The IgG titers were converted from ELISA units (EU/ml) to international units (IU/ml) using the Pertussis Antiserum (human) 1st IS-WHO International Standard 06/140 (NIBSC, UK). The datasets presented in S2 Table can also be found in our recent publication [22]. (DOCX) [file pone.0219255.s002.docx]

**S2 Table. Seroprevalence of anti-PT IgG and anti-FHA IgG titers among different age groups**

| Age group (years) | n |  | Anti-PT IgG titers | |  | Anti-FHA IgG titers | |
| --- | --- | --- | --- | --- | --- | --- | --- |
|  |  |  | Mean ± SD | P value^a^ |  | Mean ± SD | P value^a^ |
| 1–2 | 20 |  | 59.3 ± 37.7 | - |  | 46.2 ± 31.6 | - |
| 3–5 | 17 |  | 19.1 ± 31.1 | < 0.001 |  | 33.3 ± 34.6 | ns |
| 6–10 | 39 |  | 35.9 ± 99.9 | < 0.001 |  | 48.3 ± 50.8 | ns |
| 11–15 | 43 |  | 21.6 ± 26.5 | < 0.001 |  | 56.0 ± 86.0 | ns |
| 16–20 | 34 |  | 17.6 ± 20.8 | < 0.001 |  | 47.0 ± 47.2 | ns |
| 21–25 | 39 |  | 17.7 ± 20.5 | < 0.001 |  | 38.9 ± 39.2 | ns |
| 26–30 | 38 |  | 16.7 ± 14.9 | < 0.001 |  | 42.8 ± 25.1 | ns |
| 31–35 | 35 |  | 18.4 ± 27.2 | < 0.001 |  | 44.0 ± 31.2 | ns |
| 36–40 | 42 |  | 16.7 ± 19.7 | < 0.001 |  | 33.4 ± 23.3 | ns |
| 41–45 | 43 |  | 29.6 ± 65.9 | < 0.001 |  | 30.8 ± 31.3 | ns |
| 46–50 | 34 |  | 30.0 ± 34.9 | ns |  | 47.6 ± 76.2 | ns |
| 51–55 | 39 |  | 15.1 ± 16.6 | < 0.001 |  | 25.0 ± 25.5 | 0.039 |
| 56–60 | 37 |  | 13.9 ± 16.1 | < 0.001 |  | 25.3 ± 22.9 | ns |

NOTE. Antibody titers are represented in IU/ml.

^a^ Dunn’s multiple comparison test; the 1–2 years age group was used as a control group for comparison. Significant at P value < 0.05, ns: not significant.
